# Murine versus human apolipoprotein E4: differential facilitation of and co-localization in cerebral amyloid angiopathy and amyloid plaques in APP transgenic mouse models

**DOI:** 10.1186/s40478-015-0250-y

**Published:** 2015-11-10

**Authors:** Fan Liao, Tony J. Zhang, Hong Jiang, Katheryn B. Lefton, Grace O. Robinson, Robert Vassar, Patrick M. Sullivan, David M. Holtzman

**Affiliations:** Department of Neurology, Hope Center for Neurological Disorders, Charles F. and Joanne Knight Alzheimer’s Disease Research Center, Washington University School of Medicine, St. Louis, MO 63110 USA; Department of Cell and Molecular Biology, The Feinberg School of Medicine, Northwestern University, Chicago, IL 60611 USA; GRECC, Durham Veterans Affairs Medical Center, Durham, NC 27710 USA; Department of Medicine (Geriatrics), Duke University Medical Center, Durham, NC 27710 USA

**Keywords:** Alzheimer’s disease, Apolipoprotein E, Amyloid plaques, Cerebral amyloid angiopathy

## Abstract

**Introduction:**

Amyloid β (Aβ) accumulates in the extracellular space as diffuse and neuritic plaques in Alzheimer’s disease (AD). Aβ also deposits on the walls of arterioles as cerebral amyloid angiopathy (CAA) in most cases of AD and sometimes independently of AD. Apolipoprotein E (apoE) ɛ4 is associated with increases in both Aβ plaques and CAA in humans. Studies in mouse models that develop Aβ deposition have shown that murine apoE and human apoE4 have different abilities to facilitate plaque or CAA formation when studied independently. To better understand and compare the effects of murine apoE and human apoE4, we bred 5XFAD (line 7031) transgenic mice so that they expressed one copy of murine apoE and one copy of human apoE4 under the control of the normal murine apoE regulatory elements (5XFAD/apoE^m/4^).

**Results:**

The 5XFAD/apoE^m/4^ mice contained levels of parenchymal CAA that were intermediate between 5XFAD/apoE^m/m^ and 5XFAD/apoE^4/4^ mice. In 5XFAD/apoE^m/4^ mice, we found that Aβ parenchymal plaques co-localized with much more apoE than did parenchymal CAA, suggesting differential co-aggregation of apoE with Aβ in plaques versus CAA. More importantly, within the brain parenchyma of the 5XFAD/apoE^m/4^ mice, plaques contained more murine apoE, which on its own results in more pronounced and earlier plaque formation, while CAA contained more human apoE4 which on its own results in more pronounced CAA formation. We further confirmed the co-aggregation of mouse apoE with Aβ in plaques by showing a strong correlation between insoluble mouse apoE and insoluble Aβ in PS1APP-21/apoE^m/4^ mice which develop plaques without CAA.

**Conclusions:**

These studies suggest that both murine apoE and human apoE4 facilitate differential opposing effects in influencing Aβ plaques versus CAA via different co-aggregation with these two amyloid lesions and set the stage for understanding these effects at a molecular level.

**Electronic supplementary material:**

The online version of this article (doi:10.1186/s40478-015-0250-y) contains supplementary material, which is available to authorized users.

## Introduction

The accumulation of amyloid β (Aβ) into plaques is one of the pathological hallmarks of Alzheimer’s disease (AD) [13]. The vast majority of patients diagnosed with AD also have cerebral amyloid angiopathy (CAA), deposition of Aβ on the cerebral vessels [16]. Some individuals develop CAA in the absence of AD [3]. CAA often associates with hemorrhagic lesions, ischemic lesions, encephalopathy, and dementia [39].

The strongest known genetic risk factor for late onset AD is the ε4 allele of apolipoprotein E (apoE), while the ε2 allele is protective [7, 8, 33]. Human apoE4 carriers have higher amyloid plaque load as well as greater amounts of CAA [12, 20, 29]. ApoE influences deposition of Aβ in plaques and CAA likely through some common mechanisms affecting Aβ clearance and aggregation. For example, apoE4 slows down Aβ clearance and leads to higher Aβ concentration [6]. This could further increase Aβ accumulation in both plaques and CAA. ApoE co-localizes with both amyloid plaques and CAA [21, 35, 37], and blocking apoE/Aβ binding with a non-fibrillogenic synthetic peptide Aβ_12-28p_ reduces plaque load as well as CAA [27, 40]. In addition, amyloid precursor protein (APP) transgenic mice lacking apoE have a marked reduction of fibrillar Aβ deposition and no CAA, suggesting that apoE facilitates Aβ deposition in both lesions [2, 11, 15].

Out of 299 amino acids, human apoE4 shares only 70 % homology with mouse apoE. Using APP transgenic (Tg) mice that develop Aβ deposition, it has been shown that mouse apoE is overall more amyloidogenic than any of the human apoE isoforms [9]. In addition, mouse apoE appears to be more prone to lead to parenchymal plaque formation while human apoE4 is more prone to lead to CAA formation in mice that generate wild type human Aβ peptide [10, 24]. This conflicting pattern suggests that apoE may affect plaques and CAA deposition via a different mechanism(s). This difference is not likely caused by differential overall Aβ clearance rates since a change in Aβ clearance and consequent concentration would probably have the same impact on both plaques and CAA. Another possibility is that this difference is mediated by differential co-aggregation of apoE with Aβ in parenchymal plaques or CAA. In this study, we asked whether mouse apoE vs human apoE4 differentially 1) lead to either parenchymal plaque formation versus CAA and 2) co-aggregate with Aβ in plaques and CAA by quantifying their degree of co-localization with plaques and CAA when they are expressed in the same brain at the same level.

Here we utilized APP Tg mice carrying one copy of endogenous mouse apoE and one copy of apoE4 (APP/apoE^m/4^). In this model, different apoE proteins interact with Aβ under an identical in vivo microenvironment. Therefore, the interactions between apoE and Aβ will depend on the intrinsic properties to each apoE isoform.

We first quantified the amyloid plaque and CAA load in 8-10 month old 5XFAD/apoE^m/m^, 5XFAD/apoE^m/4^ and 5XFAD/apoE^4/4^ mice and verified that apoE4 strongly facilitated amyloid deposition in CAA, and that mouse apoE facilitated parenchymal plaque deposition. We then assessed 5XFAD/apoE^m/4^ mice and found that in the presence of both mouse apoE and human apoE4, there was an intermediate level of CAA as compared to either 5XFAD/apoE^m/m^ or 5XFAD/apoE^4/4^ mice. In 5XFAD/apoE^m/4^ mice, mouse apoE co-localized with parenchymal plaques to a significantly greater extent while apoE4 co-localized with CAA to a significantly greater extent. Further, in 85 day old APPPS1-21/apoE^m/4^ mice, which have plaques without CAA, insoluble Aβ was strongly correlated with insoluble mouse apoE but not apoE4. The data suggest that the type of apoE dictates whether apoE will lead to greater plaque versus CAA and that differences in apoE sequence and co-aggregation with plaques versus CAA likely leads to this difference. Understanding the molecular basis for this difference will lead to insights into disease pathogenesis that may have future treatment implications.

## Materials and methods

### Animals

5XFAD mice, line Tg7031 on a C57/B6XSJL background (gift from Dr. Robert Vassar at Northwestern University) co-express the KM670/671NL, I716V, and V717I mutations in human APP (695), as well as the M146L and L286V mutations in human PS1 under control of the mouse Thy1 promoter [23]. APPPS1-21 mice on a C57BL/6 J background (gift from Dr. Mathias Jucker at Hertie-Institute for Clinical Brain Research) co-express human APP with a Swedish mutation (KM670/671NL) and mutant PS1 with the L166P mutation under the control of a Thy1 promoter [25]. ApoE4 knockin mice express apoE ε4 under control of the endogenous mouse regulatory elements on a C57BL/6 J background (apoE^4/4^) [34]. 5XFAD mice carrying one copy (5XFAD/apoE^m/4^) or two copies (5XFAD/apoE^4/4^) of apoE4 were generated by breeding 5XFAD/apoE^m/m^ with apoE^4/4^ mice. APPPS1-21/apoE^m/4^ mice were generated by breeding APPPS1-21/apoE^m/m^ with apoE^4/4^ mice. Age-matched non-APP/apoE^m/4^ mice were littermates of the corresponding APP mice. All experimental protocols were approved by the Animal Studies Committee at Washington University.

### Tissue harvesting

The mice were perfused with ice cold PBS containing 0.3 % heparin. One hemibrain was dissected and flash-frozen on dry ice and then stored at -80 °C for biochemical assays. The other hemibrain was fixed in 4 % paraformaldehyde for histological study. Before staining, serial coronal sections at 50 μm thickness were collected using a freezing sliding microtome (Leica).

### X-34 staining

Quantitative analysis of fibrillar amyloid deposition was performed on 8-10 month old 5XFAD/apoE^m/m^, 5XFAD/apoE^m/4^ and 5XFAD/apoE^4/4^ mice as previously described [18]. Briefly, three sections per mouse (Bregma, -1.4 mm caudal to Bregma, -2.0 mm caudal to Bregma) were stained with X-34 and then scanned using a Nanozoomer slide scanner (Hamamatsu Photonics). Images were exported with NDP viewer (Hamamatsu Photonics), converted to grayscale, thresholded to highlight positive staining of plaques or CAA, and analyzed using ImageJ (National Institutes of Health). The average area covered by X-34 from the 3 sections/mouse was used to represent each mouse. The quantification was performed by an investigator who was blinded for the genotype of the animals.

### Immunohistochemistry

Brain sections from 10-month-old 5XFAD/apoE^m/4^ or 85-day-old APPPS1-21/apoE^m/4^ mice were co-stained for fibrillar amyloid, mouse apoE, and apoE4 using X-34, anti-mouse apoE monoclonal antibody HJ6.3-Alexa Fluor 568 (generated in-house) and anti-human apoE monoclonal antibody HJ15.7-Alexa Fluor 488 (generated in-house), respectively. For quantification of apoE/CAA or apoE/plaque co-staining in 10-month-old 5XFAD/apoE^m/4^ mice, brain sections were imaged using a Zeiss LSM 5 PASCAL system coupled to a Zeiss Axiovert 200 M confocal microscope. Three sections from each mouse were used and five fields containing CAA and five fields containing plaques on each section were imaged. Images were thresholded for corresponding colors and the degree of co-localization was analyzed with ImageJ. Percent area of CAA or plaque covered by mouse apoE or apoE4 was calculated and the average from the 3 sections/mouse was used to represent each mouse. For quantification of apoE/plaques in 85-day-old APPPS1-21/apoE^m/4^ mice, which were at the initiation of plaque deposition, all the plaques in the cortical area were counted under Nikon Eclipse 80i fluorescent microscope and the apoE co-staining status for each individual X-34 plaque was recorded. The average of 3 sections from each mouse was used to represent each mouse.

### Tissue lysate ELISA

Brain cortices from 85-day-old APPPS1-21/apoE^m/4^ and apoE^m/4^ mice were sequentially homogenized with cold PBS, 1 % triton-X 100, and 5 M guanidine buffer in the presence of 1X protease inhibitor mixture (Roche). Aβ_40_, Aβ_42_, mouse apoE, and apoE4 in each fraction were measured by ELISA. For Aβ_40_ or Aβ_42_ ELISA, anti-Aβ_35-40_ HJ2 (generated in-house) or anti-Aβ_37-42_ HJ7.4 (generated in-house) was used as the capture antibody, and anti-Aβ_13-28_ HJ5.1-biotin (generated in-house) as the detecting antibody [4]. For mouse apoE ELISA, plates were coated with HJ6.2 (in-house generated) at a concentration of 10 μg/ml in carbonate coating buffer at 4 °C overnight. After blocking with 2 % BSA in PBS at 37 °C for 1 h, the samples were loaded on the plates and incubated overnight at 4 °C. Then the plates were incubated in 300 ng/ml HJ6.8-biotin (generated in-house) at 37 °C for 1.5 h. Followed by a incubation in 1:10000 Streptavidin Poly-HRP40 Conjugate (Fitzgerald) at room temperature for 1.5 h, the plates were developed using Super Slow ELISA TMB (Sigma) and read on a Bio-Tek Synergy 2 plate reader at 650 nm. The standard curve was mouse apoE purified from mouse astrocytes conditioned medium using a polyclonal antibody (Calbiochem). Human apoE ELISA shared the same protocol as mouse apoE ELISA except for the antibodies were different. For human apoE ELISA, HJ15.6 (generated in-house) at a concentration of 10 μg/ml was used as the capture antibody and 150 ng/ml HJ15.4-biotin (generated in-house) was used as detecting antibody. Recombinant apoE4 (Leinco) was used as the standard for human apoE ELISA.

### Data analysis

Statistical analyses were performed using GraphPad Prism (GraphPad Software, version 5.0). All data were analyzed using ANOVA, One-way ANOVA with repeated measures, student *t*-test or paired *t- * test as indicated in the respective figure legends. Sample sizes were specified in the respective figure legends. Data were expressed as mean ± S.E.M. unless otherwise specified.

## Results

### ApoE4 shifts parenchymal Aβ deposition from plaques to CAA in the 5XFAD mice

**Fig. 1:**
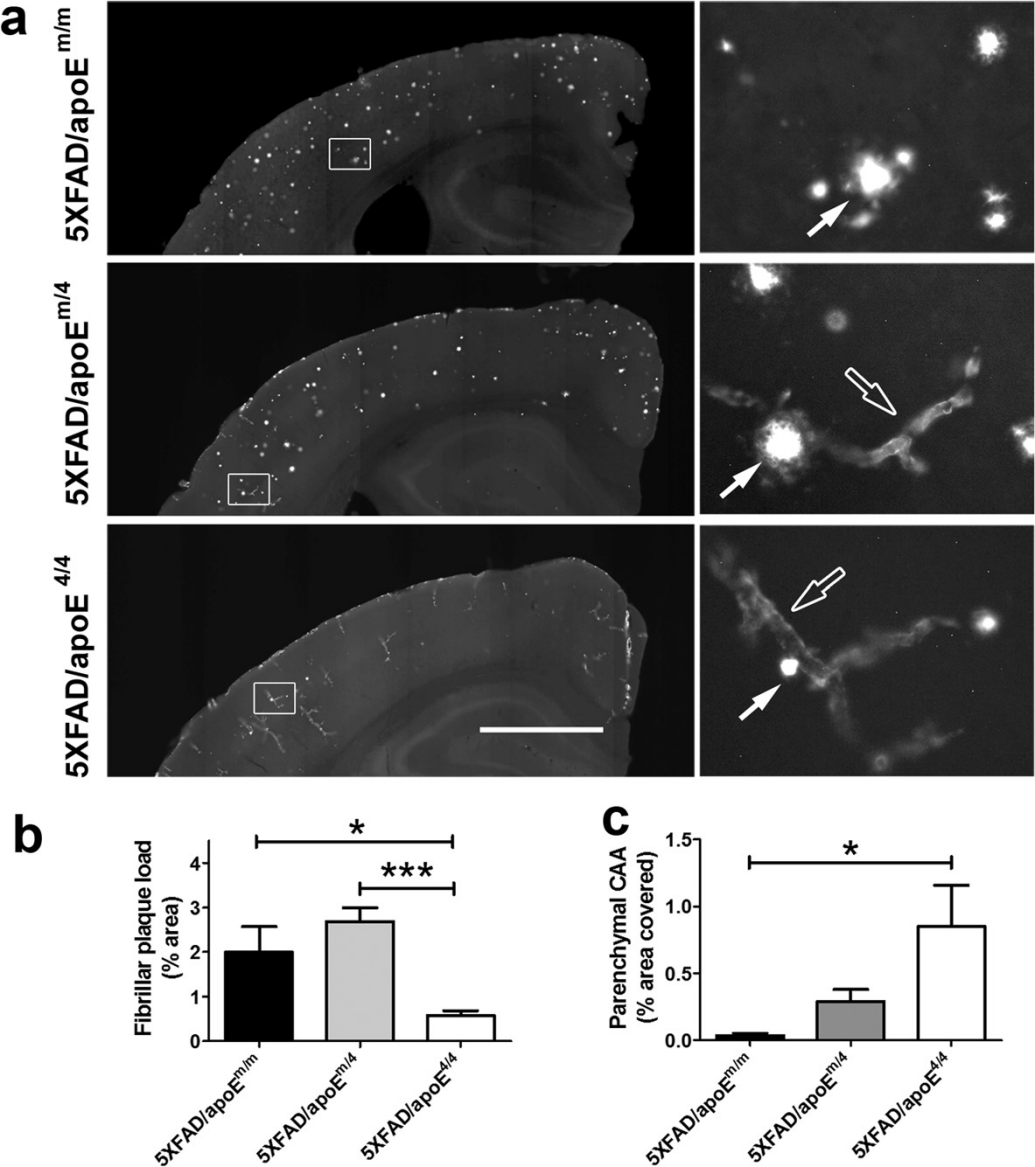
ApoE4 shifted parenchymal Aβ deposition from plaques to parenchymal CAA in the 5XFAD mice. 8 ~ 10 months old 5XFAD/apoE^m/m^, 5XFAD/apoE^m/4^ and 5XFAD/apoE^4/4^ mice were stained with X-34. **a** Representative brain sections with CAA (empty arrows) and plaques (solid arrows). Scale bar, 1 mm. The right panel is the high power magnification of the area labeled in the squares in the corresponding left-side images. **b** The % area covered by parenchymal fibrillar plaques in the cortex. **c** The % area covered by parenchymal CAA quantified in the cortex (*n* = 3-9/group; *p < 0.05, One-way ANOVA followed by Tukey post-test)

Plaque deposition begins in 5XFAD (line 7031) on mouse apoE background (5XFAD/apoE^m/m^) at the age of 4 months. When 5XFAD mice were bred onto an apoE4 background (5XFAD/apoE^4/4^), plaque deposition began at the age of 5 months (Additional file 1: Figure S1). To further determine the effect of replacing mouse apoE with apoE4 on Aβ plaques and CAA, we stained fibrillar amyloid with X-34 in 8-10 month old 5XFAD/apoE^m/m^, 5XFAD/apoE^m/4^ and 5XFAD/apoE^4/4^ mice (Fig. 1a). The amount of CAA on the blood vessels within the brain parenchyma (parenchymal CAA) and amyloid plaques were quantified. There is minimal parenchymal CAA compared to amyloid plaques in 5XFAD/apoE^m/m^ animals (Fig. 1b, c). With the introduction of one copy of apoE4 (5XFAD/apoE^m/4^), the plaque load did not change while the CAA tended to increase (Fig. 1b, c). With two copies of apoE4 (5XFAD/ apoE^4/4^), there was a significant reduction of plaque load (Fig. 1b) and a significant elevation of CAA (Fig. 1c) as compared to 5XFAD/apoE^m/m^ mice.

### Plaques contain more mouse apoE while parenchymal CAA contain more apoE4 in 5XFAD/apoE^m/4^ mice

**Fig. 2:**
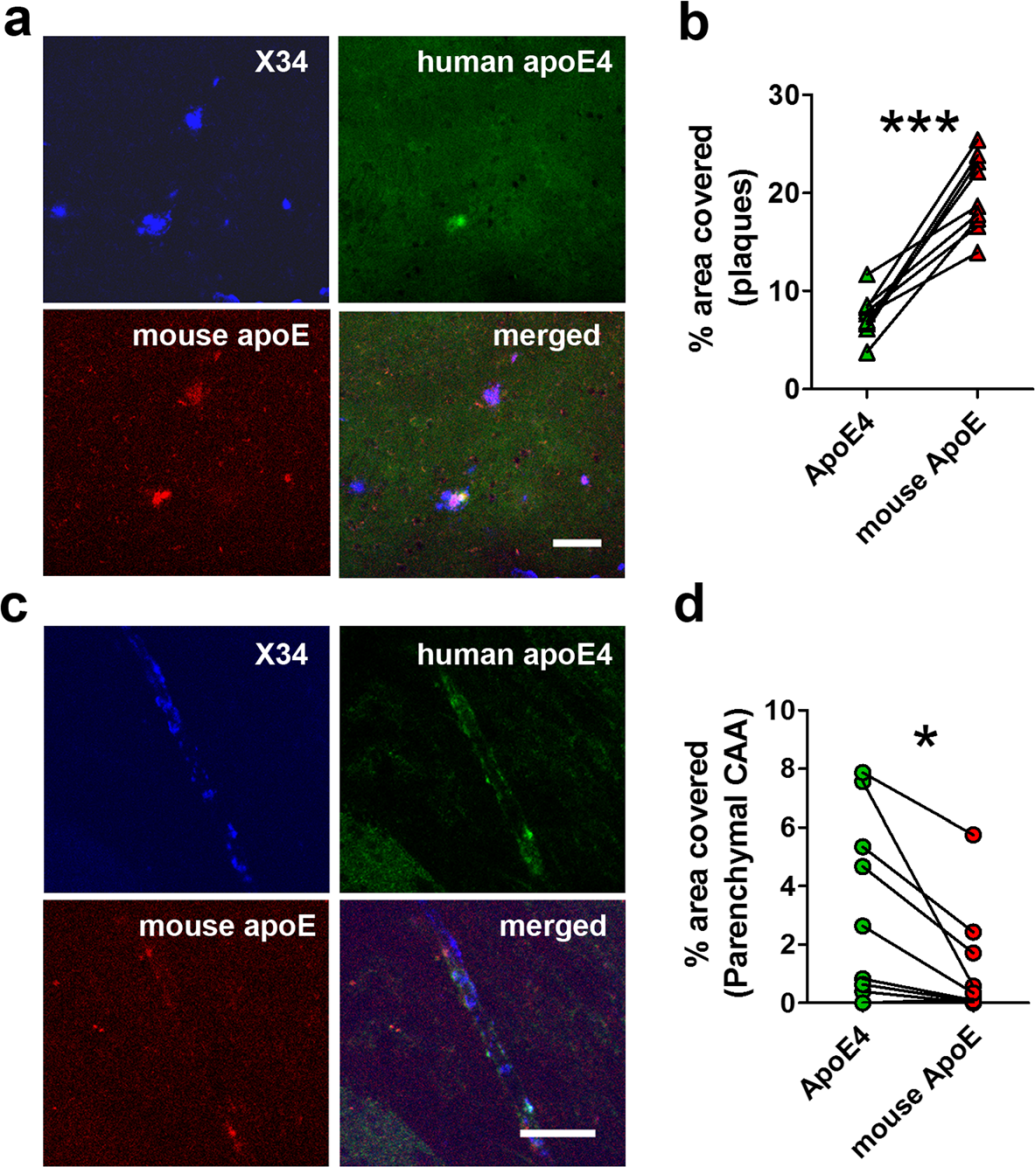
Co-localization of mouse apoE and apoE4 in CAA or plaques within the same brain parenchyma in 5XFAD/apoE^m/4^ mice. 10-month-old 5XFAD/apoE^m/4^ mice were co-stained with HJ6.3-Alexa 568 for mouse apoE, HJ15.7-Alexa 488 for apoE4, and X-34 for fibrillar amyloid. **a**-**b** Representative images of co-staining for mouse apoE and apoE4 in the plaques and % area of plaque covered by different apoE. **c**-**d** Representative images of co-staining for mouse apoE and apoE4 in parenchymal CAA and % area of parenchymal CAA covered by different apoE. Values connected by lines were measured from the same animals (*n* = 9/group; *p < 0.05, ***p < 0.001, paired *t*-test)

To determine whether the mouse apoE and apoE4 facilitate plaque or CAA differently via differential co-aggregation with Aβ in plaques and CAA, we examined the amount of apoE4 or mouse apoE co-localized with plaques and CAA. We co-stained amyloid deposition with an anti-mouse apoE specific antibody HJ6.3 (Additional file 2: Figure S2) and an anti-human apoE specific antibody HJ15.7 (Additional file 2: Figure S2) in 10-month-old 5XFAD/apoE^m/4^ brains (Fig. 2). We first quantified co-localization of each form of apoE in relation to plaques and CAA. Within brain parenchyma, both apoE4 (3.33 ± 1.04 % in CAA vs 7.49 ± 0.71 % in plaques, *n* = 9, *p* < 0.05, paired *t*-test) and mouse apoE (1.23 ± 0.63 % in CAA vs 19.89 ± 1.30 % in plaques, *n* = 9, *p* < 0.001, paired *t*-test) exhibited less co-localization in parenchymal CAA as compared to plaques. However, the amount of each form of apoE localized in the same lesion was different. Parenchymal plaques contained more mouse apoE immunoreactivity than apoE4 (Fig. 2a, b), whereas parenchymal CAA contained more apoE4 immunoreactivity than mouse apoE (Fig. 2c, d).

**Fig. 3:**
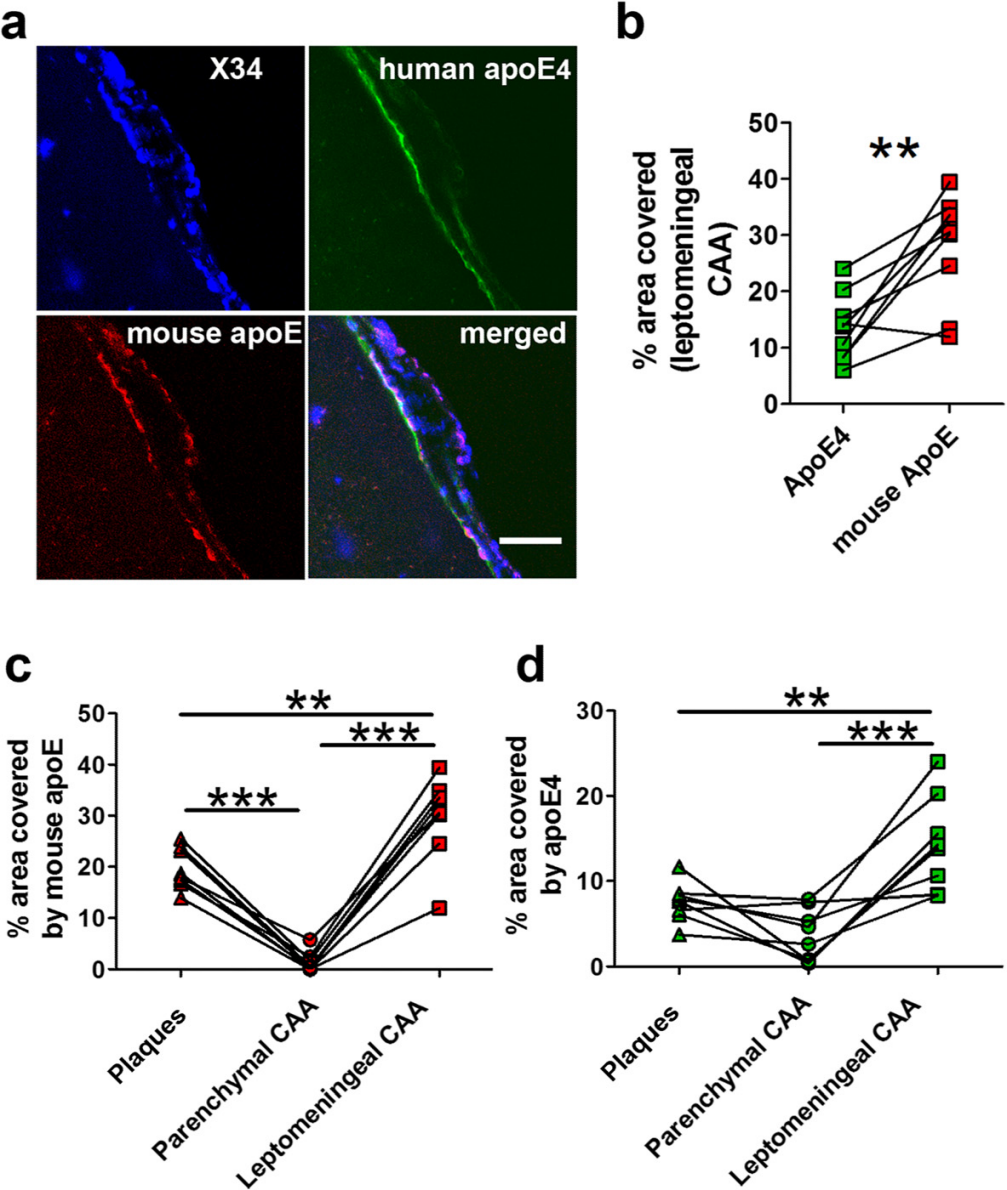
Comparison between apoE co-localization in parenchymal and leptomeningeal CAA in the same 5XFAD/apoE^m/4^ brains. Brain sections from 10 months old 5XFAD/apoE^m/4^ animals were co-stained with HJ6.3-Alexa 568 for mouse apoE, HJ15.7-Alexa 488 for human apoE4, and X-34 for fibrillar amyloid. **a**-**b** Representative images of co-staining for mouse apoE and apoE4 in the leptomeningeal CAA and % area of leptomeningeal CAA covered by different apoE (*n* = 9/group). Scale bars, 50 μm. **c** % area of plaque, parenchymal and leptomeningeal CAA covered by mouse apoE. **d** % area of plaque, parenchymal and leptomeningeal CAA covered by human apoE4. Values connected by lines were measured from the same animals (*n* = 8/group; **p < 0.01, ***p < 0.001, One-way ANOVA repeated measures)

Next, we compared the co-localization of different apoE within parenchymal CAA (Fig. 2c) and the CAA on leptomeningeal vessels (leptomeningeal CAA, Fig. 3a). Interestingly, both mouse apoE (Fig. 3c) and apoE4 (Fig. 3d) exhibited significantly more co-localization with leptomeningeal CAA as compared to parenchymal CAA. In addition, while apoE4 co-localized more than mouse apoE within parenchymal CAA (Fig. 2d), leptomeningeal CAA co-localized with less apoE4 as compared to mouse apoE (Fig. 3b). For the mouse apoE to human apoE4 ratio, the ratios were similar in plaques and leptomeningeal CAA while parenchymal CAA had the lowest ratio (Additional file 3: Figure S3).

### Mouse apoE and Aβ levels are highly correlated in plaques in APPPS1-21/apoE^m/4^ cortices

**Fig. 4:**
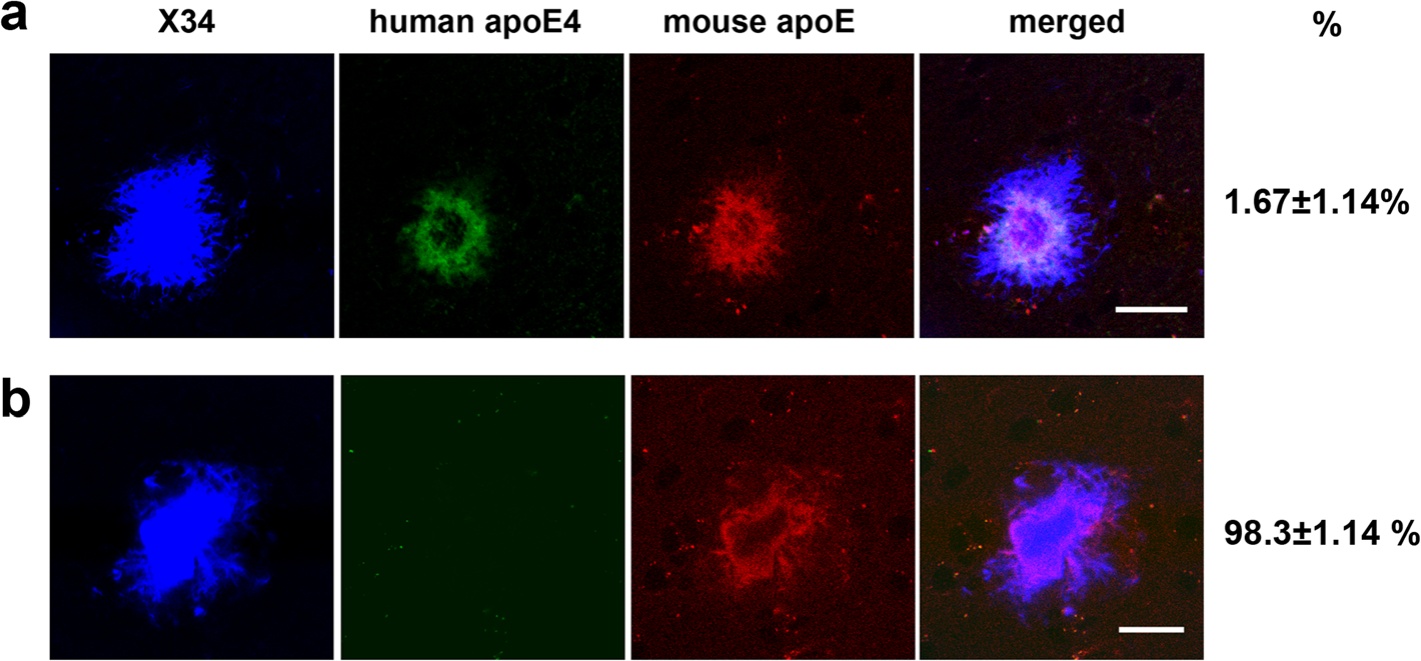
Co-localization of mouse apoE and apoE4 in plaques within the same brain parenchyma in APPPS1-21/apoE^m/4^ mice. Brain sections from 85-day-old APPPS1-21/apoE^m/4^ animals (*n* = 7) were co-stained with HJ6.3-Alexa 568 for mouse apoE, HJ15.7-Alexa 488 for human apoE4, and X-34 for fibrillar amyloid. **a** Representative images and the % of plaques containing both mouse apoE and apoE4 (scale bar, 20 μm). **b** Representative images and the % of plaques containing only mouse apoE (scale bar, 20 μm)

To verify the differential co-aggregation of mouse vs. human apoE4 with Aβ plaques, we utilized another APP Tg mouse model, APPPS1-21. We first co-stained amyloid deposition with specific anti-mouse apoE antibody HJ6.3 and specific anti-human apoE antibody HJ15.7 in 85-day-old APPPS1-21/apoE^m/4^ mice (Fig. 4). We found that CAA was absent in the APPPS1-21/apoE^m/4^ cortex at this age, probably due to the low Aβ_40_/Aβ_42_ ratio in the APPPS1-21 mouse model [25]. Interestingly, all (100 %) of the plaques contained mouse apoE (Fig. 4a, b); whereas only a very small percentage (1.67 ± 1.14 %) of plaques also contained apoE4 (Fig. 4a). There was no plaque that only contained apoE4.

**Fig. 5:**
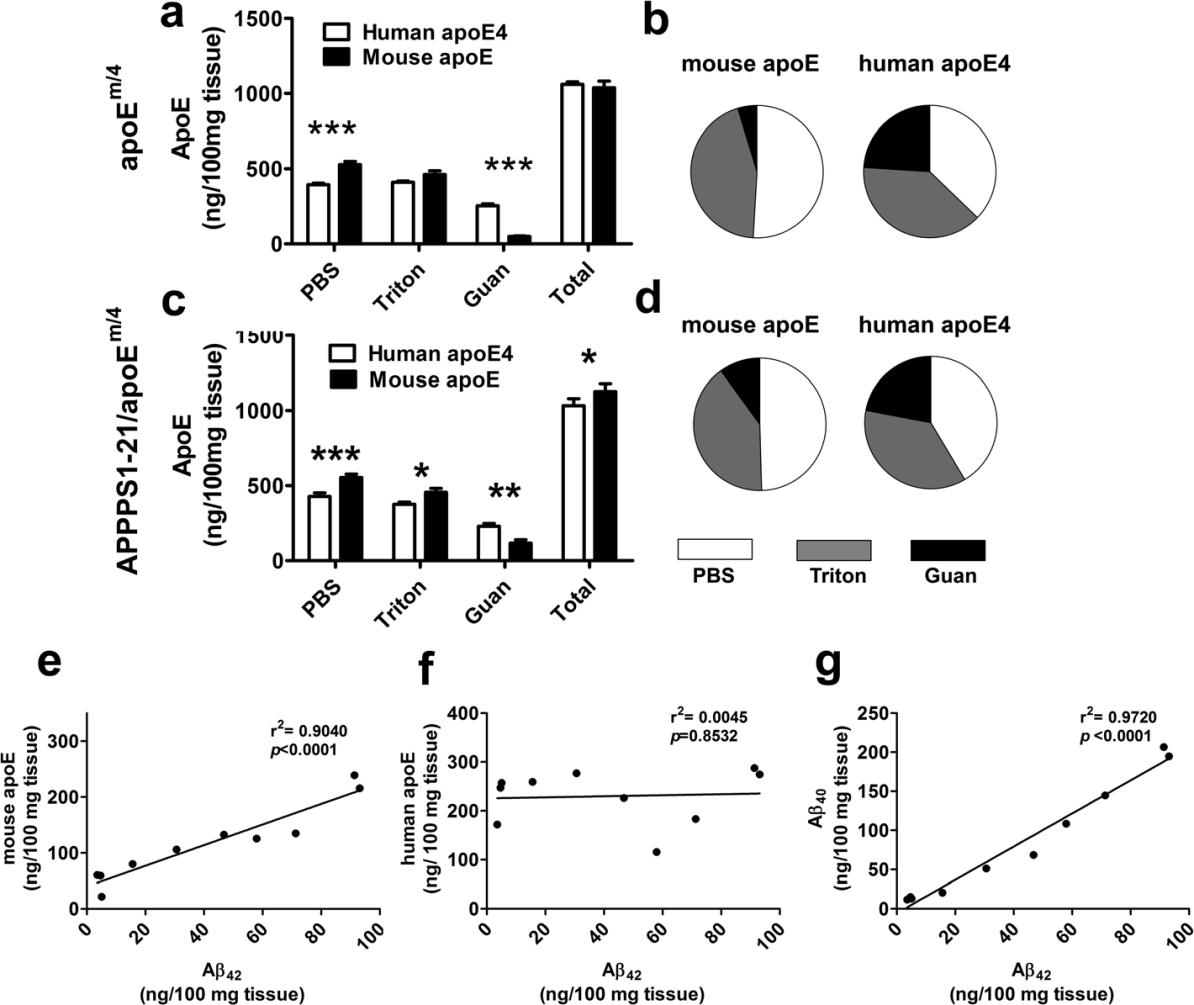
ApoE levels in 85-day-old apoE^m/4^ and APPPS1-21/apoE^m/4^ brains. The cortices were homogenized in PBS, followed by 1 % Triton X-100 and 5 M guanidine and apoE levels were measured by ELISA. **a**-**b** Absolute concentrations and fractional distribution of mouse apoE and apoE4 in apoE^m/4^ mice (*n* = 10). **c**-**d** Absolute concentrations and fractional distribution of mouse apoE and apoE4 in APPPS1-21/apoE^m/4^ mice (*n* = 10; *p < 0.05; **p < 0.01; ***p < 0.001, Two-way ANOVA for repeated measures followed by Bonferroni post-test). **e**-**g** Correlations of Aβ_42_ with mouse apoE, apoE4 and Aβ_40_ in the insoluble fraction of APPPS1-21/apoE^m/4^ cortices (*n* = 10)

To assess the level of mouse and human apoE in cortical tissue lysates of the same APPPS1-21/apoE^m/4^ mice, we developed both a mouse and a human apoE specific ELISA (Additional file 4: Figure S4). We then performed a 3-step sequential extraction of APPPS1-21/apoE^m/4^ cortex using PBS, 1 % Triton X-100 and 5 M guanidine, and measured apoE levels in these three fractions. Tissue from age-matched non-APP transgenic apoE^m/4^ mice were also analyzed as controls to see the basal levels of apoE in the absence of amyloid plaques. In the non-APP transgenic, apoE^m/4^ mice, we found that the mouse apoE and apoE4 levels were similar in the sum of the three fractions (Fig. 5a). However, the fractional distributions of different apoE were very different. For mouse apoE, 50.9 ± 1.1 % was present in the PBS soluble fraction and only 4.7 ± 0.4 % was present in the insoluble (guanidine) fraction (Fig. 5b). ApoE4 was more equally distributed in 3 fractions (Fig. 5b). The PBS soluble fraction contained significantly more (*p* < 0.001) mouse apoE than apoE4 (Fig. 5a), while the insoluble fraction contained significantly more (*p* < 0.001) apoE4 than mouse apoE (Fig. 5a).

In APPPS1-21/apoE^m/4^ cortex which contained amyloid plaques, apoE4 absolute levels (Fig. 5c) or fractional distribution (Fig. 5d) were unaltered as compared to that in apoE^m/4^ cortices. For mouse apoE, the absolute levels were unaltered in the PBS and 1 % Triton fraction of APPPS1-21/apoE^m/4^ cortices as compared to that in apoE^m/4^. However, the mouse apoE in the insoluble fraction of APPPS1-21/apoE^m/4^ mice was significantly higher than that in apoE^m/4^ mice (117.3 ± 21.7 *vs* 48.8 ± 4.4 ng/100 mg tissue for APPPS1-21/apoE^m/4^ and apoE^m/4^, respectively; *p* < 0.01, Student *t*-test). As a result, the percentage of insoluble mouse apoE was increased in APPPS1-21/apoE^m/4^ mice as compared to that in apoE^m/4^ mice (10.0 ± 1.6 % vs 4.7 ± 0.4 % for APPPS1-21/apoE^m/4^ and apoE^m/4^, respectively; *p* < 0.01, Student *t*-test; Fig. 5b, d). The increase of insoluble mouse apoE is very likely due to co-aggregation of mouse apoE with Aβ in the plaques, which did not occur to a significant extent with apoE4. To verify this possibility, we performed correlation analysis of insoluble Aβ and apoE levels in the same sample in the cortices of these APPPS1-21/apoE^m/4^ mice. Mouse apoE demonstrated a strong correlation with insoluble Aβ_42_ (Fig. 5e), in agreement with the observation that mouse apoE highly co-localized with plaques by co-staining. In contrast, apoE4, which is poorly co-localized with plaques, demonstrated no correlation with Aβ_42_ (Fig. 5f). As expected, insoluble Aβ_40_ strongly correlated with insoluble Aβ_42_ in APPPS1-21/apoE^m/4^ mice (Fig. 5 g).

## Discussion

*APOE* genotype is the strongest genetic risk factor for late-onset AD and CAA. ApoE4 is associated with increases in both plaque burden and CAA in humans relative to the other human apoE isoforms [17]. ApoE likely influences deposition of Aβ in plaques and CAA through some common mechanisms affecting Aβ clearance and aggregation. Interestingly, previous studies in mouse models of amyloid deposition have shown that murine apoE is significantly more amyloidogenic than human apoE isoforms, including apoE4 [9, 41]. Despite the fact that it is more amyloidogenic, previous studies showed that apoE4 led to greater CAA than mouse apoE. In the current study, we showed that mouse apoE and human apoE4 were each dominant when present in the same brain (Fig. 1): mouse apoE promoted plaques while apoE4 promoted CAA. Overall, these findings suggest that differences in the inherent properties/structures of each form interact with Aβ in different ways leading to differential co-aggregation of Aβ in parenchymal plaque versus CAA. In addition, we found that 5XFAD/apoE^m/4^ have an intermediate level of CAA as compared to either 5XFAD/apoE^m/m^ or 5XFAD/apoE^4/4^ mice. This is important as it is consistent with human data in which it has been found that apoE4, in a dose-dependent fashion, is associated with greater parenchymal CAA [22, 30, 42].

To investigate the co-aggregation of different apoE in plaques and CAA, we assessed co-localization of apoE and amyloid in these two lesions in the same brain. Our data clearly demonstrated that within the brain parenchyma, the degree to which apoE co-localizes with Aβ in plaques or CAA was associated with its ability to facilitate the corresponding lesion. Aβ plaques contained more mouse apoE which facilitates plaque deposition (Fig. 2b), while parenchymal CAA contained more apoE4 which facilitates the formation of parenchymal CAA (Fig. 2d). In addition, the fact that parenchymal plaques contained more apoE (regardless of whether it is mouse apoE or apoE4) also suggested that apoE interacted with Aβ in plaques and CAA differently.

While most apoE present in physiological fluids such as CSF may not complex with monomeric Aβ [36], there is no question that once Aβ aggregates in the brain parenchyma or in CAA in the form of fibrils, apoE is then found co-aggregated with amyloid. However, data quantifying the amount of individual apoE isoforms co-depositing with Aβ in plaques or CAA are lacking. Although in PDAPP/apoE^4/4^ mice, the co-localization of apoE with Aβ plaques was higher than that in PDAPP/apoE^3/3^ and PDAPP/apoE^2/2^ mice [1], the different Aβ concentration and oligomerization states in individual mice with different apoE genotypes could influence the results. In the current study, the co-localization of different forms of apoE with different amyloid lesions within brain parenchyma was compared in the same mice carrying one copy of each apoE isoform and expressing each copy under the same regulatory elements at the same level (5XFAD/apoE^m/4^). Thus, the Aβ environment was identical for both apoE isoforms and the data provided definitive in vivo confirmation that mouse apoE and human apoE4 differentially facilitate specific types of amyloid deposition at least in part by co-aggregating with them differentially. The different % area covered by mouse apoE vs. human apoE4 observed here is likely not an artifact caused by the performance of apoE antibodies since plaques co-localized more with mouse apoE while parenchymal CAA co-localized more with apoE4. Furthermore, this co-localization of apoE and Aβ in plaques was verified by correlating Aβ with different apoE in the insoluble fraction of APPPS1-21/apoE^m/4^ mice. Interestingly, while our current data and previous studies using APP transgenic mice generating normal human Aβ [10, 24] suggested that apoE4 redistributed Aβ deposition to CAA as compared to mouse apoE, a previous study using mice expressing human Dutch/Iowa (E22Q/D23N) mutant Aβ showed an opposite pattern [38]. In transgenic mice (Tg-SwDI) that accumulate human Dutch/Iowa (E22Q/D23N) mutant Aβ, both human apoE3 and apoE4 strongly shifted the Aβ deposition from CAA into plaques [38]. Unlike in general AD populations where apoE4 is strongly associated with increased plaques and CAA, in humans with the rare Dutch mutation, apoE4 genotype was not correlated with plaques or CAA [5]. The Dutch mutation resides within amino acids 12-28 of Aβ peptide, a domain which appears to be required for interaction with apoE [33]. Taken together, our data and previous studies suggested that apoE modifies Aβ pathology through interaction with Aβ aggregates.

Since mouse apoE and apoE4 in 5XFAD/apoE^m/4^ brains were exposed to identical Aβ conditions, the co-aggregation of different apoE with Aβ in plaques or CAA was determined by the intrinsic properties of different apoE such as their binding preference to a certain species of Aβ or their concentration. CAA contains a higher Aβ_40_/Aβ_42_ ratio than do plaques [14] although Aβ_42_ is required to “seed” CAA [19]. The observation that apoE4 better co-localized with parenchymal CAA than plaques was not likely due to its higher binding preference to Aβ_40_ over Aβ_42_. If this were the case, we should expect to see even more apoE4 present than mouse apoE in the leptomeningeal CAA since the ratio of Aβ_40_/Aβ_42_ is even higher in leptomeningeal CAA than that in parenchymal CAA [26]. However in leptomeningeal CAA, we observed less apoE4 than mouse apoE. To see whether apoE levels play a role in the different co-aggregation, we measured apoE levels in cortical lysates of apoE^m/4^ and APPPS1-21/apoE^m/4^ mice. The total levels of mouse apoE and apoE4 in brain lysates were similar (Fig. 5) in apoE^m/4^ mice but their fractional distribution was different. In general there was more mouse apoE in the PBS soluble fraction while there was more human apoE4 in the insoluble fraction in the non-APP/apoE^m/4^ mice. This suggests an inherent difference in the biochemical properties of mouse apoE vs. human apoE4. It is possible that the conformation of mouse apoE in the PBS soluble fraction localized in parenchyma in such a way to interact with Aβ seeds that forms plaques as compared to apoE4 resulting in its precipitation into plaques to a greater extent. It is also possible that the structure of apoE4 may result in its localization to a greater extent in the vasculature, enabling it to interact to a greater extent with Aβ seeds that form CAA.

When leptomeningeal CAA and parenchymal CAA were compared, we found that 1) leptomeningeal CAA contained much higher apoE than did parenchymal CAA, and 2) leptomeningeal CAA contained more mouse apoE while parenchymal CAA contained more apoE4. These observations suggested that apoE might be differently involved during the formation of CAA in blood vessels of different locations.

## Conclusion

 Understanding how apoE influences the development of parenchymal plaques versus CAA is important. While this study does not provide the molecular basis for why mouse apoE and human apoE4 result in differential plaques versus CAA, it demonstrates that apoE is a major determinant of where Aβ deposits given that the Aβ in the in vivo microenvironment is the same. Therefore, studying the differences in sequence (30 % difference in sequence) and structure between mouse and human apoE that result in these differences could provide important insights. For example, understanding the structural variations could provide new insight into how to block or influence the apoE/Aβ interaction. In addition, treatment with some anti-Aβ antibodies has resulted in humans in the complication of amyloid-related imaging abnormalities either with edema or hemorrhage. This complication is far more frequent in apoE4 positive individuals [31, 32]. It was also observed that Aβ immunotherapy was associated with redistribution of apoE from cortical plaques to cerebral vessel walls, mirroring the altered distribution of Aβ [28]. Understanding the basis for the differential effects of apoE on plaques vs. CAA might provide important insights into this phenomenon that could lead to ways to understand and prevent it.

## Additional files

Additional file 1: Figure S1. Aβ plaque onset age in 5XFAD/apoE^m/m^ and 5XFAD/apoE^4/4^ brain. **A**β immunostaining was performed using biotinylated anti-Aβ_1–13_ monoclonal antibody HJ3.4B. **a** Brain section from 4 month old 5XFAD/apoE^m/m^ mouse. **b** Brain section from 4 month old 5XFAD/apoE^4/4^ mouse. **c** Brain section from 5 month old 5XFAD/apoE^4/4^ mouse. Scale bar, 1 mm. Plaques are indicated by arrows. (TIF 2146 kb) Additional file 2: Figure S2. Specific antibodies for mouse apoE and human apoE4 immunostaining. 5XFAD/apoE^m/m^ and 5XFAD/apoE^4/4^ brain sections were immunostained with HJ6.3B for mouse apoE and HJ15.7B for human apoE4 (scale bar, 400 μm). (TIF 1678 kb) Additional file 3: Figure S3. The ratio of mouse apoE to human apoE4 within different amyloid lesions in 10 month old 5XFAD/apoE^m/4^ brains shown in Fig. 2 and Fig. 3 (*n* = 8/group; ***, *p* < 0.001; One-way ANOVA repeated measures). (TIF 174 kb) Additional file 4: Figure S4. Specific ELISA for mouse apoE and human apoE. **a** Mouse apoE was detected by using HJ6.2 as the capture antibody and HJ6.8-biotin as the detecting antibody. **b** Human apoE was detected using HJ15.6 as the capture antibody and HJ15.4-biotin as the detecting antibody. (TIF 372 kb)
