# Supplementary figures and images for: Murine versus human apolipoprotein E4: differential facilitation of and co-localization in cerebral amyloid angiopathy and amyloid plaques in APP transgenic mouse models
Source: Acta Neuropathol Commun. 2015 Nov 10;3:70. doi: 10.1186/s40478-015-0250-y (PMC4641345; doi:10.1186/s40478-015-0250-y)

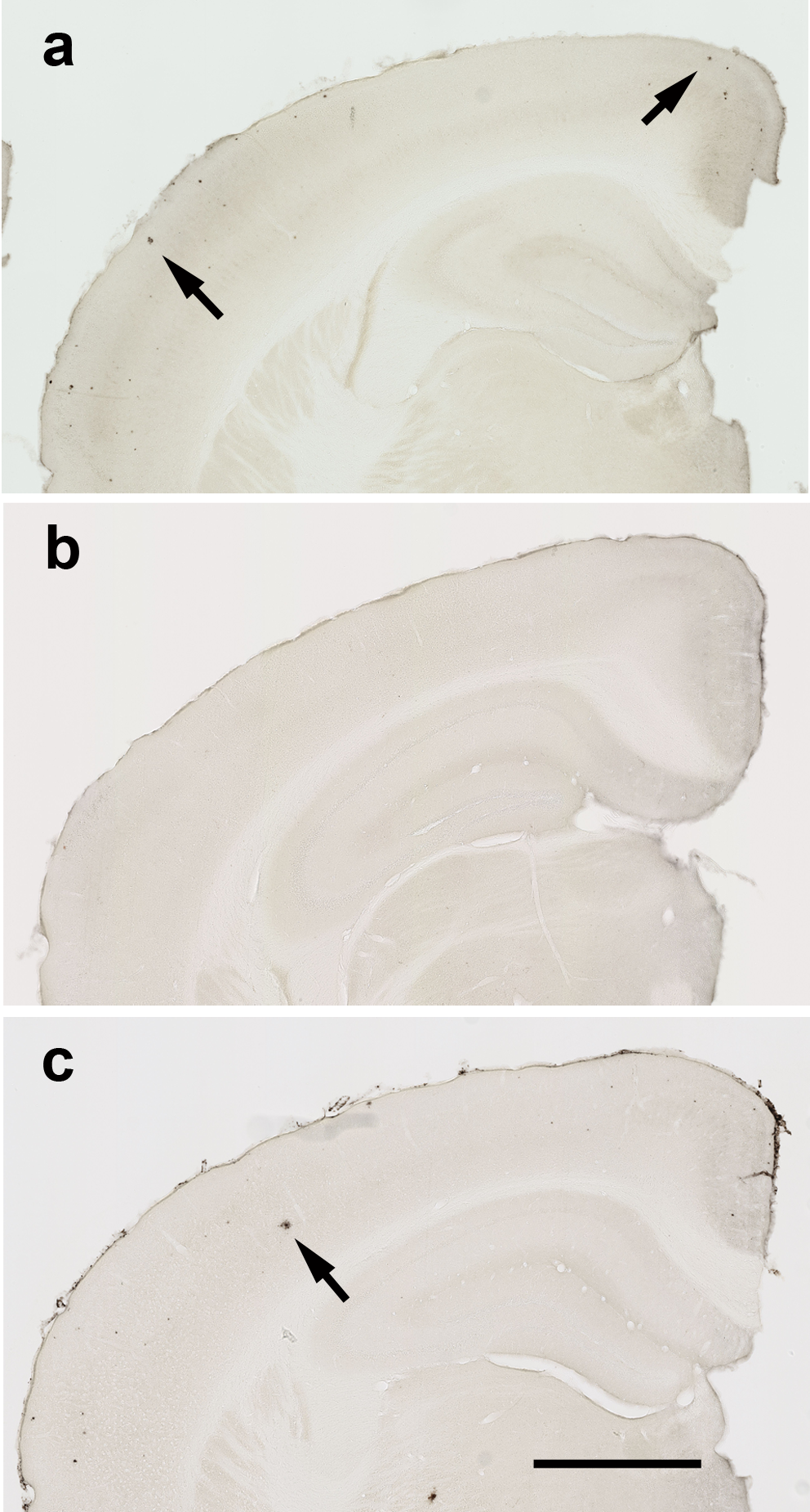

Supplement: Additional file 1: Figure S1. — Aβ plaque onset age in 5XFAD/apoEm/m and 5XFAD/apoE4/4 brain. Aβ immunostaining was performed using biotinylated anti-Aβ1–13 monoclonal antibody HJ3.4B. a Brain section from 4 month old 5XFAD/apoEm/m mouse. b Brain section from 4 month old 5XFAD/apoE4/4 mouse. c Brain section from 5 month old 5XFAD/apoE4/4 mouse. Scale bar, 1 mm. Plaques are indicated by arrows. (TIF 2146 kb) [file 40478_2015_250_MOESM1_ESM.tif]

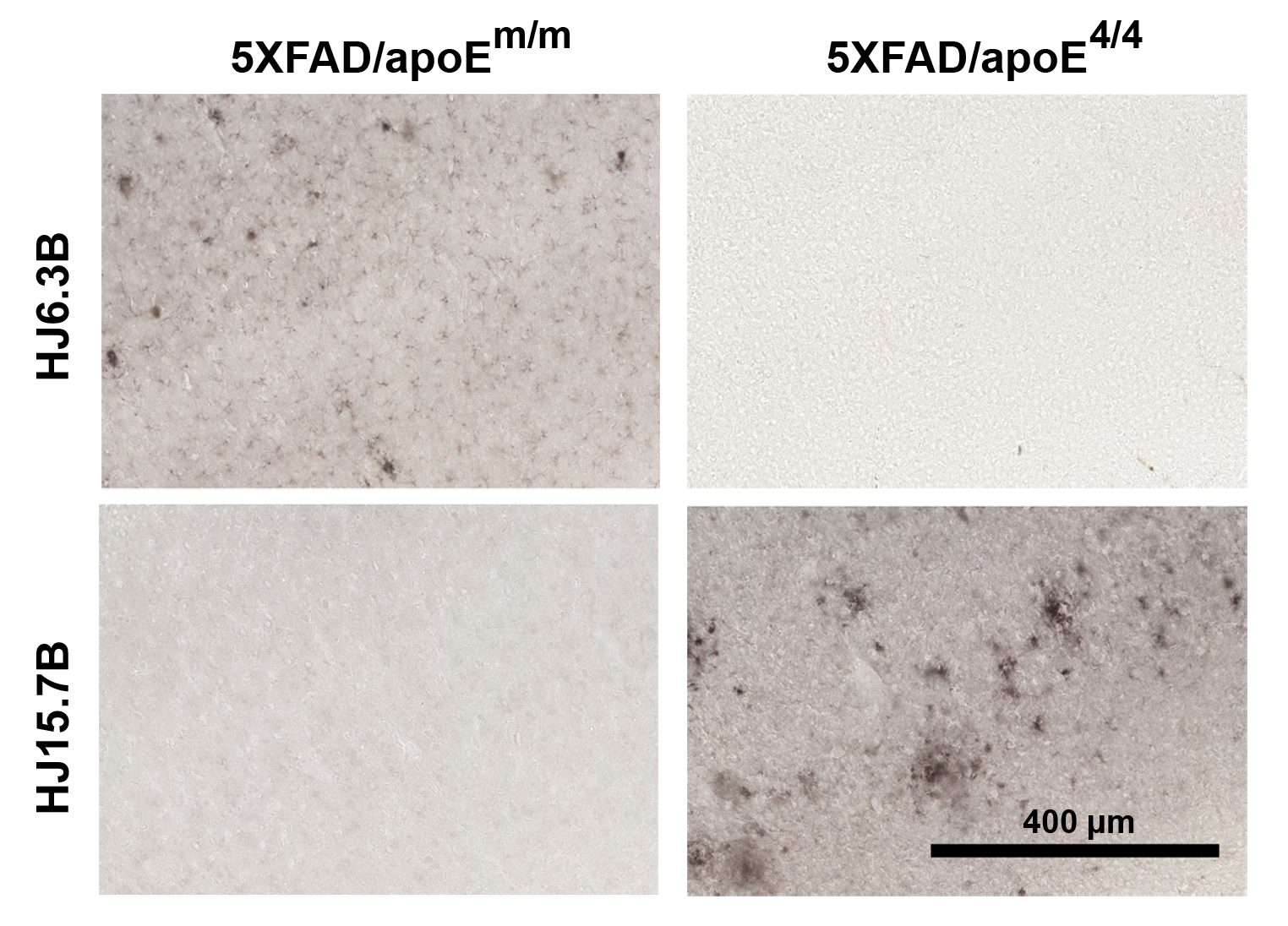

Supplement: Additional file 2: Figure S2. — Specific antibodies for mouse apoE and human apoE4 immunostaining. 5XFAD/apoEm/m and 5XFAD/apoE4/4 brain sections were immunostained with HJ6.3B for mouse apoE and HJ15.7B for human apoE4 (scale bar, 400 μm). (TIF 1678 kb) [file 40478_2015_250_MOESM2_ESM.tif]

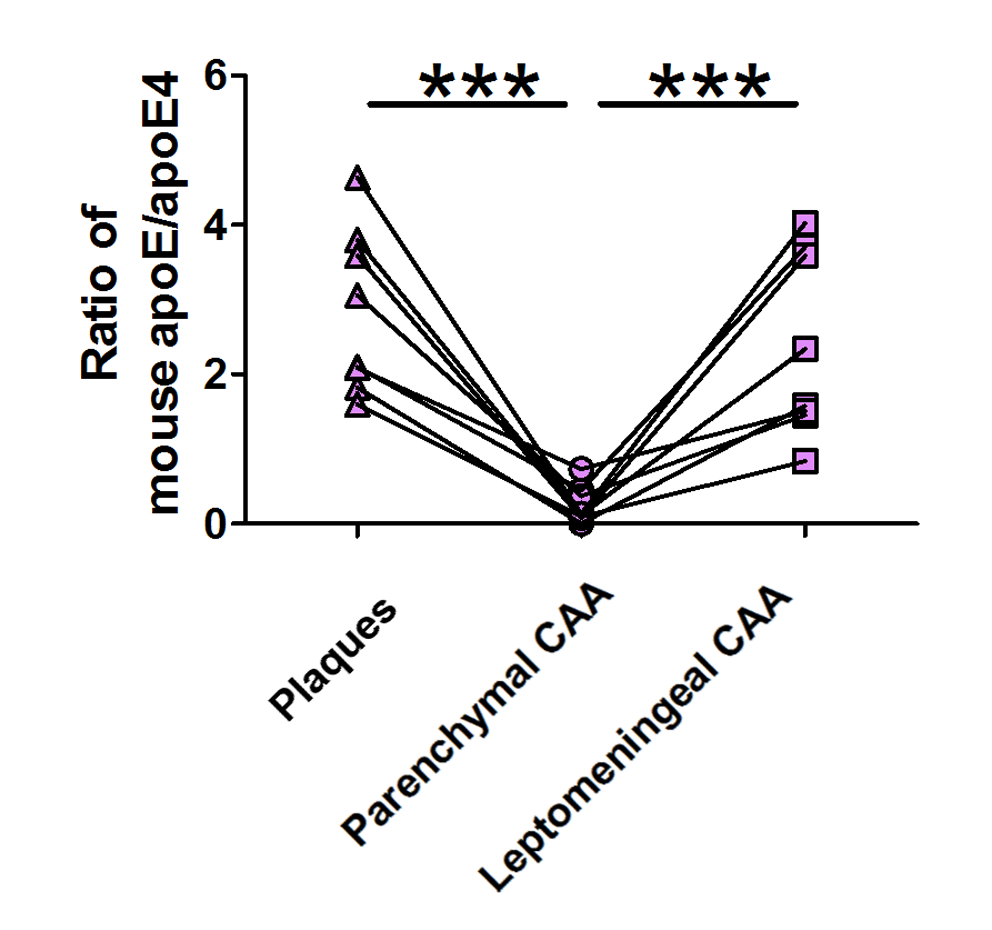

Supplement: Additional file 3: Figure S3. — The ratio of mouse apoE to human apoE4 within different amyloid lesions in 10 month old 5XFAD/apoEm/4 brains shown in Fig. 2 and Fig. 3 (n = 8/group; ***, p < 0.001; One-way ANOVA repeated measures). (TIF 174 kb) [file 40478_2015_250_MOESM3_ESM.tif]

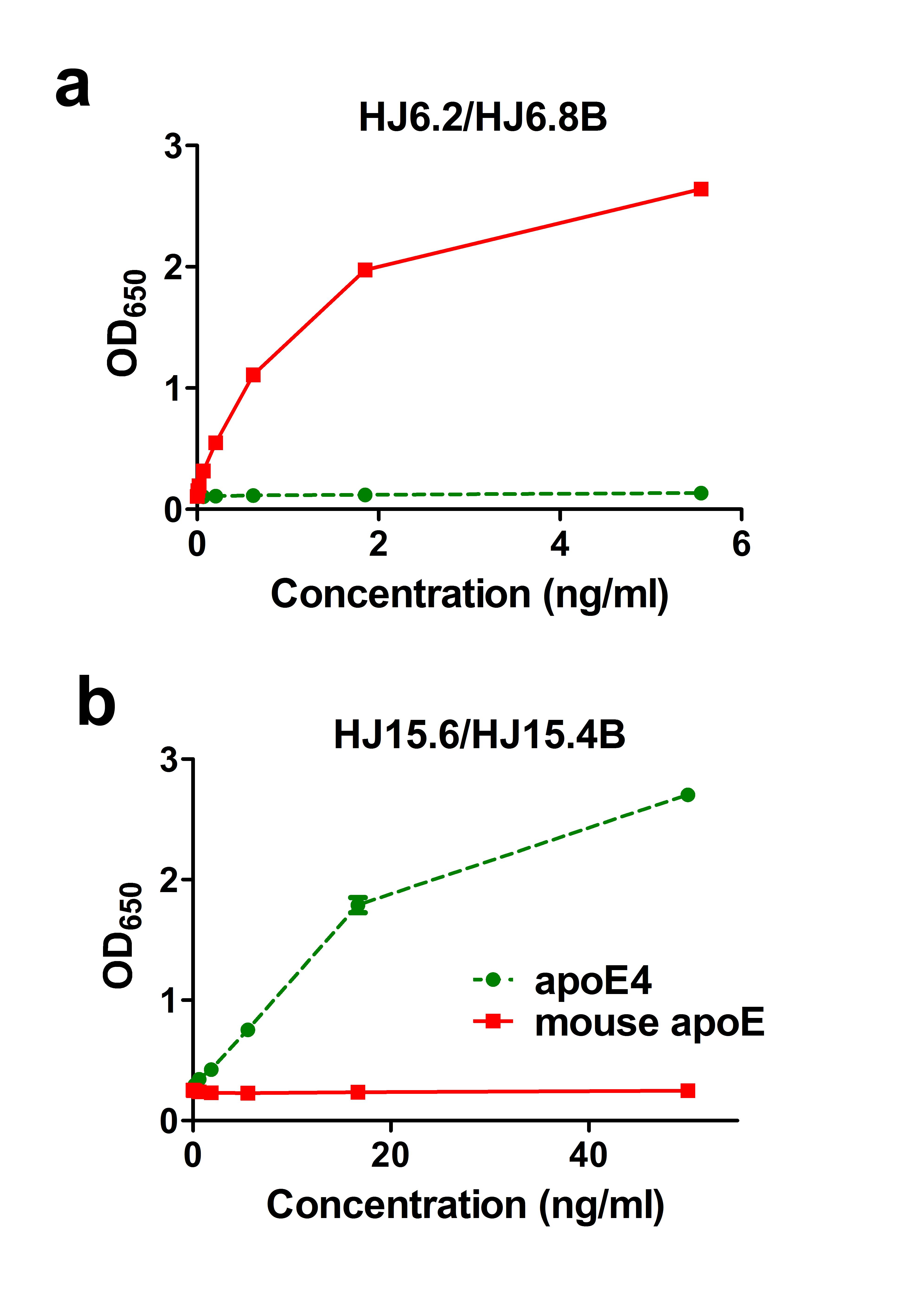

Supplement: Additional file 4: Figure S4. — Specific ELISA for mouse apoE and human apoE. a Mouse apoE was detected by using HJ6.2 as the capture antibody and HJ6.8-biotin as the detecting antibody. b Human apoE was detected using HJ15.6 as the capture antibody and HJ15.4-biotin as the detecting antibody. (TIF 372 kb) [file 40478_2015_250_MOESM4_ESM.tif]
